# Fermented Wild Chicory (*Cichorium intybus* L.) Leaves as a Promising Food Ingredient: Phytochemical Profile and Antioxidant Potential

**DOI:** 10.3390/foods15203694

**Published:** 2026-10-17

**Authors:** Elena Tomassi, Nafiou Arouna, Mattia Brun, Alessandra Bertoli, Laura Pucci

**Affiliations:** 1 Institute of Agricultural Biology and Biotechnology, National Research Council, 56124 Pisa, Italy; elena.tomassi@ibba.cnr.it (E.T.); nafiouarouna@cnr.it (N.A.); m.brun@studenti.unipi.it (M.B.); 2 Department of Pharmacy, University of Pisa, 56126 Pisa, Italy

**Keywords:** *Cichorium intybus* L., lactic acid fermentation, *Lactiplantibacillus plantarum*, bioactive compounds

## Abstract

Wild chicory (*Cichorium intybus* L.) is a spontaneous, widely distributed Mediterranean plant traditionally used in both food and herbal medicine, owing to its high content of bioactive compounds with antioxidant activity. Lactic acid fermentation is increasingly explored as a mild technological strategy to modulate the phytochemical and functional profile of vegetable matrices, but its effect on wild chicory leaves remains largely uncharacterised. In this study, fresh wild chicory leaves were subjected to controlled fermentation using inoculum suspensions containing 10^6^ cells/mL *Lactiplantibacillus plantarum*, either alone or in co-culture with *Saccharomyces cerevisiae* (10^3^ cells/mL), for 24 and 48 h at 37 °C. Fermentation with *L. plantarum*, alone or in co-culture, rapidly and significantly lowered the pH from approximately 5.5 to 4.3 within 24 h. A significant effect of time was observed for total flavonoid content, DPPH, FRAP, and ORAC, but not for total phenolic content. Averaged across treatments, flavonoid content decreased following fermentation, DPPH showed a transient increase at 24 h, FRAP was significantly lower at 48 h than at baseline, and ORAC increased at both fermented time points. Descriptive UPLC-DAD analysis of wild leaves revealed differences between unfermented and fermented sample for 24 h. Chicoric acid was detected only in the unfermented samples, whereas caffeic and 3,4-*O*-caffeoylquinic acids were detected in the fermented samples. Among flavonoids, kaempferol-3-*O*-glucoside, rutin, and luteolin were also detected, whose relative abundance varied following fermentation. These findings highlight distinct changes in the phytochemical profile and *in vitro* antioxidant capacity of wild chicory leaf extracts following fermentation, and support further investigation of fermented material as a potential food ingredient.

## 1. Introduction

Wild chicory (*Cichorium intybus* L.) is a perennial herbaceous plant native to Europe and Asia and now naturalised across most temperate regions worldwide [1,2]. Beyond its long history as a leafy vegetable, wild chicory leaves are increasingly recognised as a functional food ingredient owing to their content of phenolic acids, flavonoids and sesquiterpene lactones, which confer notable antioxidant and health-promoting properties [1,3]. Several studies have characterised the phenolic profile and *in vitro* antioxidant capacity of *C. intybus* leaves from different geographical origins and cultivation regimes, generally reporting a rich content of hydroxycinnamic acid derivatives (including chicoric and chlorogenic acids) and flavonoids such as luteolin, apigenin, quercetin and kaempferol glycosides [4,5,6,7].

Fermentation is one of the oldest food biotechnologies and is currently experiencing renewed scientific interest as a low-cost, low-energy strategy to modulate the nutritional and functional properties of plant-based foods [8,9]. According to the consensus definition proposed by the International Scientific Association for Probiotics and Prebiotics (ISAPP), fermented foods are foods or beverages produced through controlled microbial growth and the enzymatic conversion of food components [8]. Lactic acid bacteria (LAB), and *L. plantarum* in particular, are widely employed as starter cultures for vegetable fermentation owing to their acidifying, proteolytic and enzymatic activity, which can hydrolyse glycosylated phenolic compounds into aglycones and thereby modulate the phenolic profile and antioxidant capacity of the fermented matrix [10,11]. However, the direction and magnitude of these effects are strongly matrix- and strain-dependent: several vegetable fermentation studies report an increase in extractable phenolics and antioxidant capacity [11], while others describe negligible changes or even a reduction of the phenolic pool [12].

Fermentation of chicory has so far been investigated mainly on red chicory by-products, where inoculation with selected *L. plantarum* strains was shown to improve antiradical and antimicrobial properties [7]. To the best of our knowledge, no previous study has specifically investigated starter-driven fermentation of wild chicory leaves or compared fermentation with *L. plantarum* alone with co-culture fermentation involving *S. cerevisiae*.

The aim of the present study was therefore to evaluate the effect of controlled lactic acid fermentation of fresh wild chicory leaves with *L. plantarum*, used either alone or in co-culture with *S. cerevisiae*, on the total phenolic (TPC) and flavonoid (TFC) content and the *in vitro* antioxidant capacity assessed through three complementary tests (DPPH, FRAP and ORAC). Moreover, the phytochemical composition, upon fermentation, was assessed through UPLC-DAD in order to point out the main polar constituents belonging to different chemical classes (phenolics, hydroxycinnamic acids, and flavonoids). Overall, this approach may contribute to the valorisation of this spontaneous and currently underexploited plant as a novel fermented vegetable product.

## 2. Materials and Methods

### 2.1. Chemicals and Reagents

Folin–Ciocalteu reagent, sodium carbonate (Na_2_CO_3_), sodium hydroxide (NaOH), gallic acid, catechin, phosphate buffer, acetate buffer, 6-hydroxy-2,5,7,8-tetramethylchroman-2-carboxylic acid (Trolox), 2,2-diphenyl-1-picrylhydrazyl (DPPH), 2,4,6-tris(2-pyridyl)-s-triazine (TPTZ), sodium nitrite (NaNO_2_), aluminium chloride (AlCl_3_), ferric chloride hexahydrate (FeCl_3_·6H_2_O), ferrous sulphate heptahydrate (FeSO_4_·7H_2_O), 2,2′-azobis(2-amidinopropane) dihydrochloride (AAPH) and fluorescein were purchased from Sigma-Aldrich Inc. (Saint Louis, MO, USA). Methanol and ethanol were from VWR (Radnor, PA, USA). Rutin, luteolin, quercetin, apigenin, apigenin-7-*O*-glucoside, chlorogenic acid, caffeic acid, kaempferol, kaempferol-3-*O*-glucoside, 3-*O*-caffeoylquinic acid, 3,4-*O*-caffeoylquinic acid (Sigma-Aldrich, 99–98% purity) were used as reference compounds; UPLC-grade solvents MeOH Acetonitrile, MilliQ Waters were used to perform analytical sample preparation and UPLC-DAD analysis.

### 2.2. Plant Material

**Figure 1:**
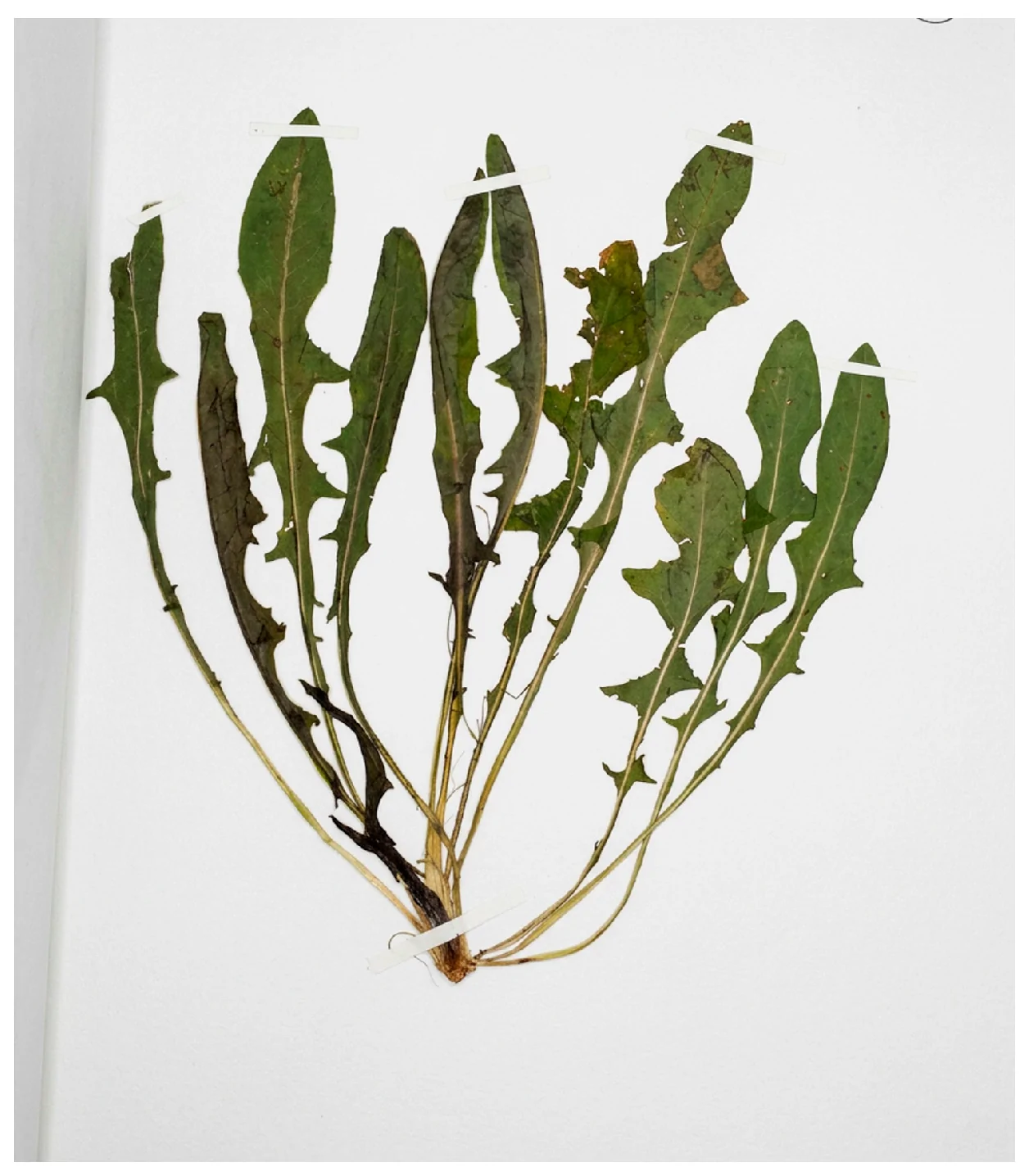
Herbarium specimens of *Cichorium intybus* L. (voucher specimens No. 69934 and 69935); Botanical Garden of the University of Pisa, collected in November 2025 at the Certosa di Calci.

Fresh leaves of wild chicory (*Cichorium intybus* L.) were collected after the flowering stage in November 2025 from plants growing spontaneously at the Certosa di Calci, in the Province of Pisa, Italy. A voucher specimen (n. 69934 and 69935) was deposited at the Botanical Garden of the University of Pisa (Figure 1). Immediately after collection, fresh leaves were selected, thoroughly washed with distilled water and used for the fermentation trials described below.

### 2.3. Starter Cultures

*S. cerevisiae* was obtained from commercial yeast (Lievital, Lesaffre Italia) and was cultivated in liquid complete medium containing 20 g/L of glucose, 10 g/L of bactopeptone and 5 g/L yeast extract (Oxoid, Basingstone, UK) at 30 °C until the stationary phase was reached. The cell concentration was determined using a Thoma counting chamber. The resulting stock suspension was then serially diluted to obtain an inoculum containing 10^3^ cells/mL for co-culture fermentation. *L. plantarum*, stored frozen in 20% glycerol, was revived in MRS broth (Oxoid) at 37 °C for 48 h. The resulting inoculum suspension contained a concentration of 10^6^ cells/mL, determined spectrophotometrically at OD600.

### 2.4. Fermentation of Cichorium intybus L. Leaves and Extraction of Bioactive Compounds

Aliquots of approximately 20 g of fresh chicory leaves were mechanically disrupted under ice-cooling and mixed with 20 mL of sterile 0.9% (*w*/*v*) NaCl solution and 5 mL of sterile double-distilled water using an Ultraturrax homogeniser (Polytron PT-MR 2100, Kinematica, Malters, Switzerland), in capped glass vessel, not hermetically sealed, and no forced aeration was applied during fermentation. Two fermentation conditions were set up in biological duplicate: (i) inoculation with *L. plantarum* alone (10^6^ cells/mL; LP), and (ii) co-inoculation with *L. plantarum* (10^6^ cells/mL) and *S. cerevisiae* (10^3^ cells/mL; LP + SC). The inocula were prepared immediately before inoculation. Samples were incubated at 37 °C under constant agitation (120 rpm) for 48 h. Approximately 10 g of fermented material was collected after 24 h (T1) and 48 h (T2) from the same fermentation vessels. The pH was measured immediately at each sampling point, and samples were stored at −80 °C prior to freeze-drying (Lyovac GT2, 4 days). An aliquot of the non-fermented starting material was freeze-dried at time zero (T0) as reference.

Freeze-dried samples were extracted in triplicate by suspending 100 mg of lyophilised material in 4 mL of 80% (*v*/*v*) ethanol, corresponding to a final sample concentration of 25 mg/mL. The extraction was carried out for 24 h at room temperature under constant orbital agitation (350 rpm) and protected from light. Following extraction, the samples were centrifuged at 2013× *g* for 10 min at 4 °C, and the resulting supernatants were collected and stored at −20 °C until the spectrophotometric assays and UPLC analyses described below.

### 2.5. Determination of pH

The pH of the fermented chicory leaves was measured at 0, 24 and 48 h using a digital pH meter (PCE-PH 26F, PCE Italia s.r.l., Lucca, Italy) equipped with a flat electrode suitable for viscous matrices, calibrated before each session with standard buffer solutions. Measurements were performed in triplicate for each sample.

### 2.6. Total Phenolic and Flavonoid Content

TPC was determined by the Folin–Ciocalteu colorimetric method [13]. Briefly, 100 µL of extract was mixed with 500 µL of Folin–Ciocalteu reagent (1:10, *v*/*v*, in water); after 5 min in the dark, 400 µL of 0.7 M Na_2_CO_3_ was added, and samples were incubated for 2 h at room temperature in the dark. Absorbance was read at 760 nm (FLUOstar Omega, BMG Labtech, software version 5.70 R2). A calibration curve was built with gallic acid in 80% methanol, and results were expressed as mg gallic acid equivalents (GAE) per g of dry weight (DW).

TFC was determined using the aluminium chloride colorimetric method [14]. To 100 µL of extract (or solvent for the blank), 400 µL of water and 30 µL of 5% NaNO_2_ were added and incubated for 5 min; 30 µL of 10% AlCl_3_ was then added (replaced by water in the blank), followed by 6 min of incubation. The reaction was neutralised with 200 µL of 1 M NaOH and 240 µL of water, and after 30 min absorbance was read at 430 nm. Catechin in methanol was used as the calibration standard, and results were expressed as mg catechin equivalents (CE) per g DW.

### 2.7. Antioxidant Capacity

The DPPH radical scavenging assay was performed following Colosimo et al. [15]. Twenty-five µL of extract was mixed with 975 µL of methanolic DPPH solution (60 µM) and incubated in the dark for 30 min; absorbance was read at 517 nm. Results were expressed as mg Trolox equivalents (TE) per g DW, using a Trolox calibration curve.

The ferric reducing antioxidant power (FRAP) assay [16] was based on the reduction of the Fe^3+^–TPTZ complex to its blue Fe^2+^ form. FRAP reagent (TPTZ 0.01 M in 0.04 M HCl, FeCl_3_·6H_2_O 0.02 M, and 0.3 M acetate buffer pH 3.6, 1:1:10 *v*/*v*/*v*) was added to the extract (967:33 µL), and absorbance was read at 593 nm at time zero and after 30 min. Results were calibrated against FeSO_4_·7H_2_O and expressed as µmol Fe^2+^ equivalents (Fe^2+^E) per g DW.

The oxygen radical absorbance capacity (ORAC) assay was performed according to Ninfali et al. [17]. Extract (100 µL) was mixed with 800 µL of fluorescein (40 nM in Tris-HCl buffer, pH 8) and 100 µL of AAPH (400 mM in 75 mM phosphate buffer, pH 7.4). Fluorescence decay, at 37 °C, was monitored (excitation 485 nm; emission 510 nm) with a total of 60 measurements recorded at 30 s intervals. Results were calculated relative to a Trolox standard curve, were expressed as µmol Trolox equivalents (TE) per g DW.

### 2.8. UPLC-DAD Analysis of Phenolic Compounds

UPLC-DAD analyses compared unfermented material T0 with samples collected after 24 h of fermentation (T1), providing a descriptive assessment of the phytochemical profile. The 24 h sampling point was selected for further phytochemical characterisation based on the overall phytochemical and antioxidant responses observed. Compared with 48 h, the shorter processing time may also offer practical advantages by reducing the duration of temperature control and agitation and, consequently, the associated energy requirements, although energy consumption was not directly assessed in this study. However, this selection does not establish an optimal fermentation time. Material from the two independent biological batches was pooled for each condition and analysed in technical triplicate. The unfermented and 24 h fermented leaf extracts, as described in Section 2.4, were filtered through a 0.45 µm PTFE membrane filter (Phenomenex, Torrance, CA, USA) prior to UPLC-DAD analysis. Analyses were performed using a Waters Acquity Arc UPLC system equipped with a Sample Manager FTN autosampler (50 µL loop), a reversed-phase XSelect HSS T3 column (150 mm × 2.1 mm, 2.5 µm, Waters, Milford, MA, USA), and a mobile-phase gradient from CH_3_CN–H_2_O 5:95 (0.1% HCOOH) to 80:20 over 11 min at a flow rate of 0.3 mL/min. Reference standards belonging to the phenolic acid, caffeoylquinic acid derivative, flavonol and flavone classes were analysed under the same experimental conditions: chicoric acid, caffeic acid, chlorogenic acid, 4-*O*-caffeoylquinic acid, 3,4-*O*-caffeoylquinic acid, apigenin, apigenin-7-*O*-glucoside, kaempferol, kaempferol-3-*O*-glucoside, luteolin, rutin and quercetin (UPLC-grade MeOH, –110 ppm). Peak identification was carried out by co-chromatography with the authentic standards and by comparing the retention times and UV spectra of reference compounds injected at the same condition. Each chromatographic profiles were recorded over a wavelength range of 200–600 nm. Quali-quantitative UPLC-DAD analysis was performed by internal standard (chloramphenicol, Rt 8.81 ± 0.36, 100 ppm, MeOH) at 270 nm (Table 1 and Table 2), according to the characteristic wavelengths reported for these standard compounds [18,19,20].

### 2.9. Statistical Analysis

Two independent fermentation batches were prepared for each treatment (*n* = 2) and sequentially sampled at 0, 24, and 48 h from the same vessels. Each sample was analysed in triplicate, and the three technical measurements were averaged prior to statistical analysis and were not treated as independent experimental units. Data are expressed as mean ± standard deviation (SD) of the independent fermentation batches. TPC, TFC, DPPH, FRAP, and ORAC data were analysed by two-way repeated-measures ANOVA, with Time (0, 24, and 48 h) as the within-subject factor and fermentation treatment (LP and LP + SC) as the between-subject factor. Main effects of Time and Fermentation treatment and their interaction were evaluated. Sphericity was assessed using Mauchly’s test, and no significant violation was detected for any parameter (*p* > 0.05). Pairwise comparisons among time points were performed using Bonferroni adjustment for multiple comparisons. UPLC-DAD data were evaluated descriptively. For each condition, material from the two independent biological batches was pooled, and analysed in triplicate. Results are expressed as mean ± SD of three technical replicates. The SD reflects analytical variability and does not estimate variability between biological batches; thus, the SD reflects analytical variability rather than variability between biological batches. Statistical analyses were performed using IBM SPSS Statistics, version 30 (IBM Corp., Armonk, NY, USA), with statistical significance set at *p* < 0.05.

## 3. Results

### 3.1. pH Changes During Fermentation

**Figure 2:**
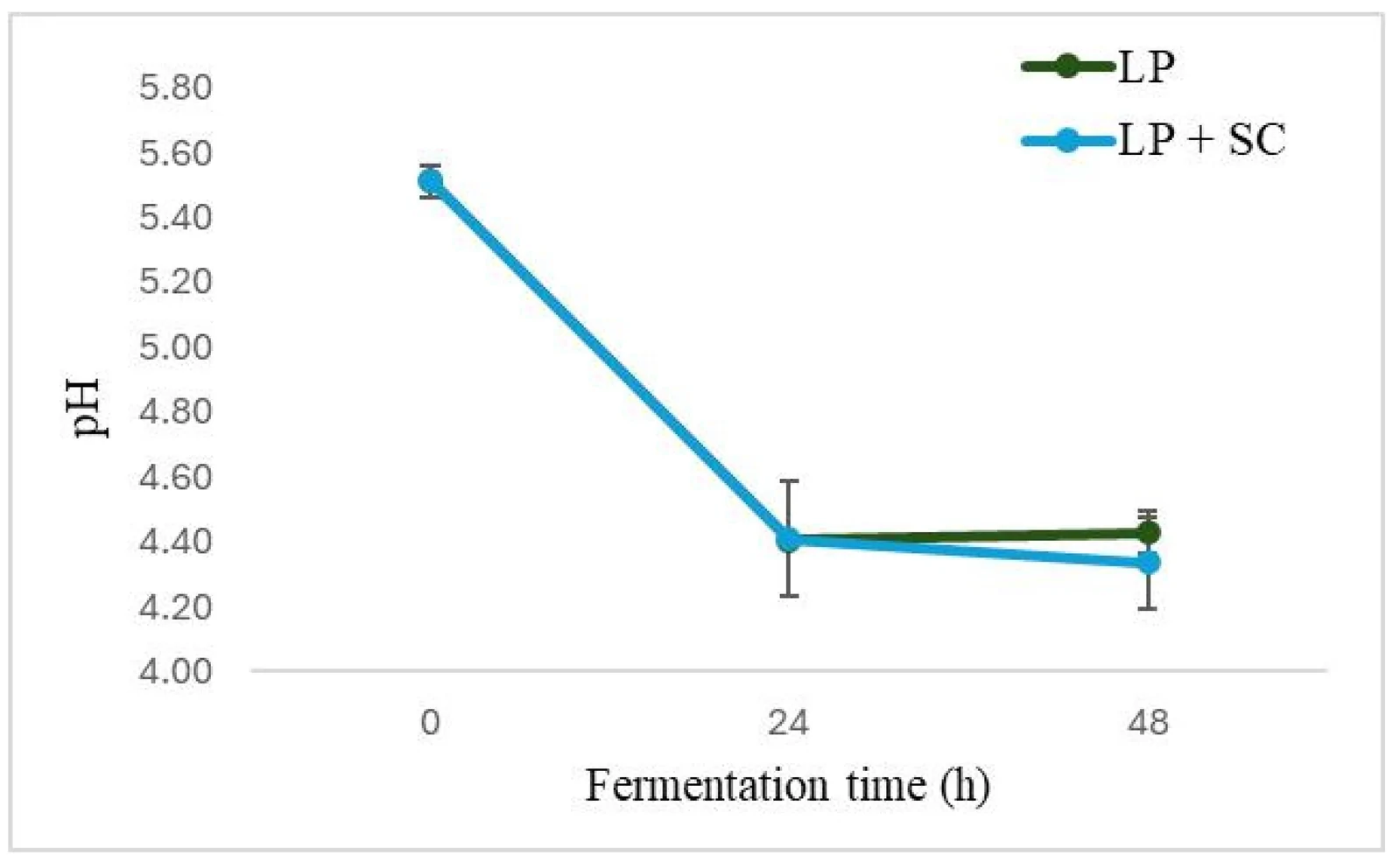
pH kinetics of wild chicory leaves fermented with *L. plantarum* (LP) alone or in co-culture with *S. cerevisiae* (LP + SC), at 0, 24 and 48 h. Data are expressed as mean ± SD. Samples represent two independent fermentation batches per treatment, each measured in technical triplicate.

At time zero, all samples showed comparable, weakly acidic pH values (~5.5), with no significant differences between the batches destined for the two inoculation conditions. Inoculation with *L. plantarum* (LP), alone or in co-culture with *S. cerevisiae* (LP + SC), produced a rapid and significant acidification of the matrix within the first 24 h (Figure 2). The pH decreased to 4.41 ± 0.18 for LP and 4.41 ± 0.01 for LP + SC at 24 h and remained essentially stable after 48 h (4.43 ± 0.14 and 4.34 ± 0.06, respectively). The decrease relative to T0 was observed for both conditions, confirming the effectiveness of the *L. plantarum* inoculum in driving a typical lactic-type fermentation and reaching a stable fermentative equilibrium within 24 h.

### 3.2. Total Phenolic and Flavonoid Content and Antioxidant Capacity

Neither fermentation treatment nor the Time × Fermentation treatment interaction was statistically significant for TPC or TFC. TPC was not significantly affected by time (F(2,4) = 2.759, *p* = 0.177). The treatment × time means are shown in Table 3. TPC was 3.795 ± 0.049 mg GAE/g at T0 for both treatments, 3.541 ± 0.371 and 3.306 ± 0.061 mg GAE/g for LP at 24 and 48 h, respectively, and 3.717 ± 0.490 and 3.439 ± 0.404 mg GAE/g for LP + SC at 24 and 48 h, respectively.

In contrast, TFC varied significantly with time (F(2,4) = 23.941, *p* = 0.006). When averaged across fermentation treatments, TFC decreased from 9.437 ± 0.000 mg CE/g at T0 to 6.994 ± 0.632 and 7.178 ± 0.883 mg CE/g at 24 and 48 h, respectively (Table 3). Both fermented time points were significantly lower than T0, whereas no significant difference was observed between 24 and 48 h.

Fermentation treatment and the time × fermentation treatment interaction were not significant for any of the antioxidant assays.

DPPH radical scavenging capacity showed a significant time effect (F(2,4) = 21.409, *p* = 0.007; Table 3). The mean at 24 h (1.095 ± 0.160 mg TE/g) was significantly higher than those at T0 (0.909 ± 0.068 mg TE/g) and 48 h (0.853 ± 0.062 mg TE/g), which did not differ significantly.

FRAP also varied with time (F(2,4) = 14.470, *p* = 0.015; Table 3). Reducing capacity was significantly lower at 48 h (27.126 ± 1.989 µmol Fe^2+^E/g) than at T0 (36.970 ± 0.000 µmol Fe^2+^E/g). The intermediate value at 24 h (33.189 ± 3.781 µmol Fe^2+^E/g) did not differ significantly from either T0 or 48 h.

ORAC showed a significant time effect (F(2,4) = 32.954, *p* = 0.003; Table 3). Values increased from 31.570 ± 4.073 µmol TE/g at T0 to 42.191 ± 1.215 and 42.003 ± 3.058 µmol TE/g at 24 and 48 h, respectively. Both fermented time points were significantly higher than T0, corresponding to increases of approximately 34% and 33%, and did not differ significantly from each other.

### 3.3. Phytochemical Profile of Unfermented and Fermented Chicory Leaves

**Figure 3:**
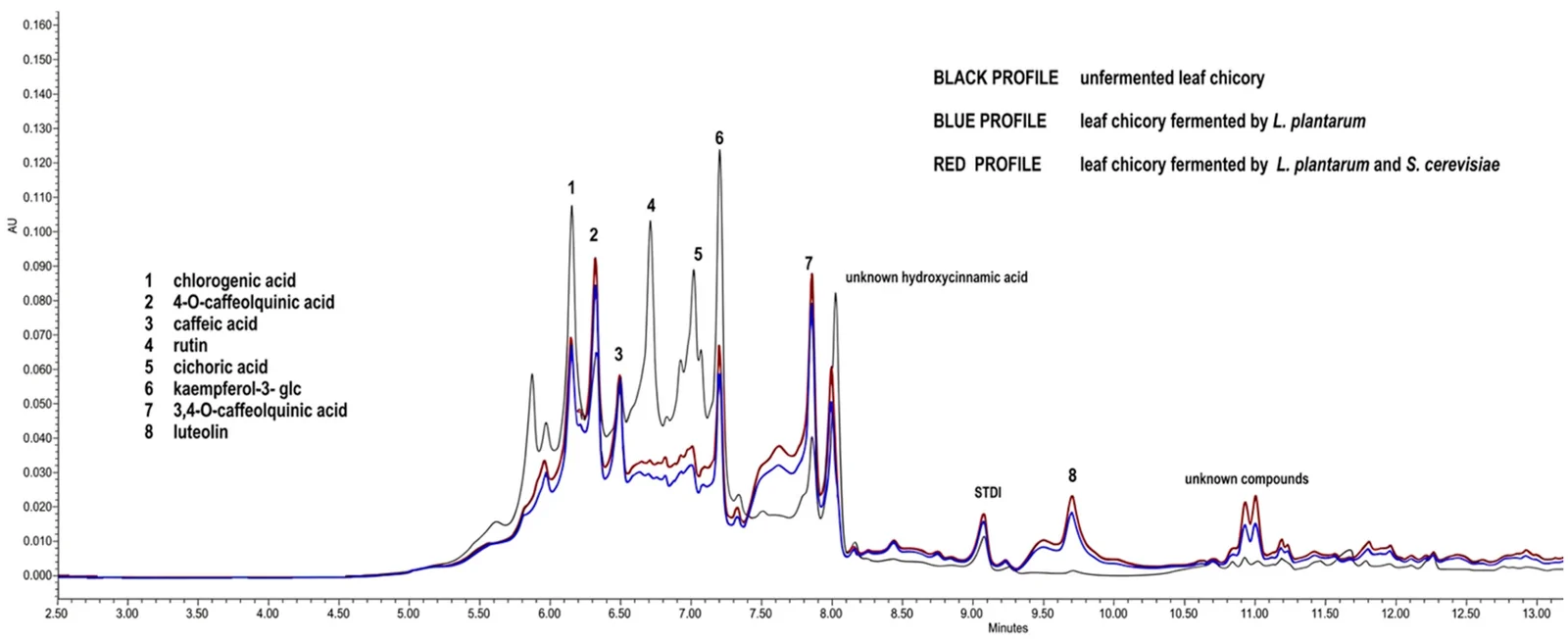
Comparison UPLC-DAD profiles of unfermented and 24 h fermented extracts of wild chicory leaves with *L. plantarum* (LP) or *L. plantarum* in co-culture with *S. cerevisiae* (LP + SC). STDI referring to Internal Standard: chloramphenicol.

Descriptive UPLC-DAD analysis revealed differences in the phytochemical profile between unfermented and 24 h fermented chicory leaf extracts (Figure 3). As shown in Table 4, among the flavonoids, kaempferol-3-*O*-glucoside and rutin were detected under the analytical conditions in the unfermented samples, at concentrations of 40.68 ± 0.01 and 66.08 ± 0.43 mg/100 g, respectively. Following fermentation, rutin was no longer detected, while kaempferol-3-*O*-glucoside decreased to 19.08 ± 0.46 mg/100 g in LP and 11.78 ± 0.02 mg/100 g in LP + SC. Luteolin was detected at low concentrations in both unfermented and LP-fermented samples (6.09 ± 0.0.01 and 7.13 ± 0.09 mg/100 g, respectively), whereas it was not detected in LP + SC samples. Fermentation also markedly affected the profile of hydroxycinnamic acids. Chlorogenic acid decreased from 66.70 ± 0.54 mg/100 g in the unfermented samples to 19.53 ± 0.36 and 4.92 ± 0.30 mg/100 g after LP and LP + SC fermentation. Conversely, 3,4-*O*-caffeoylquinic acid and caffeic acid were detected only in traces levels in the unfermented samples, at concentrations below the lower limit of the linearity range established for the corresponding reference standards. Both compounds were detected after fermentation, reaching 14.03 ± 0.09 and 7.77 ± 1.37 mg/100 g in LP and 22.10 ± 1.04 and 16.25 ± 1.64 mg/100 g in LP + SC samples, respectively. Overall, fermentation was associated with a marked reshaping of the phenolic profile, characterized by the depletion of some compounds originally detected in the unfermented samples and the appearance of others following fermentation.

## 4. Discussion

The pH decreased to approximately 4.3–4.4 within 24 h, in both inoculated treatments, is consistent with the acidifying activity reported for *L. plantarum* in vegetable matrices, where the production of organic acids rapidly decreases the pH and contributes to microbiological stability [7,21,22]. The limited changes between 24 and 48 h indicate that most of the observed acidification occurred during the first 24 h. This trend was observed in both the *L. plantarum* and *L. plantarum + S. cerevisiae* treatments, suggesting comparable acidification patterns under the experimental conditions applied. However, pH measurements alone cannot provide information on microbial safety or on the growth, viability, and metabolic activity of the inoculated microorganisms, as microbial enumeration and organic acid analyses were not performed. In addition, uninoculated controls developed visible mould growth by 24 h and were excluded from subsequent analyses. Consequently, comparisons with the time-zero reference could not fully distinguish starter-associated changes from those related to incubation or endogenous microbial activity.

The overall stability of TPC across fermentation times and types of inoculum contrasts with reports describing a significant reduction of phenolics during LAB fermentation of other vegetable substrates, which has been attributed to microbial changes in phenolic compounds [23]. However, it is consistent with studies in which selected starter cultures promoted the release or transformation of phenolic compounds without resulting in a net change in the total phenolic pool [24,25,26]. This apparent heterogeneity indicates that the effect of lactic fermentation on TPC is strongly matrix-, microorganism-, and process-dependent. The results showed time-dependent changes in TFC and antioxidant responses, without detecting an effect of starter composition or a treatment x time interaction. TFC was lower in all fermented samples than T0. This pattern may reflect differences in the flavonoid profile and/or extractability. Glycosidase-mediated transformations have been reported in LAB-fermented plant matrices [24,25,26,27], but neither enzyme activity nor a specific transformation pathway was investigated in the present study.

The pattern observed across the three antioxidant assays illustrates how the response of a fermented matrix can differ substantially depending on the underlying reaction mechanism. DPPH activity showed a transient increase in the LP treatment at 24 h, followed by a decrease at 48 h, whereas the LP + SC treatment remained close to or slightly below the unfermented control. The FRAP capacity, based primarily on electron-transfer reactions, decreased significantly upon fermentation, particularly after 48 h in both inoculated conditions. Such variability is consistent with studies reporting that antioxidant responses depend on the plant substrate, microbial strain and fermentation conditions, including fermentation duration [14,28]. The higher ORAC values indicate enhanced in *in vitro* peroxyl-radical scavenging capacity in the fermented extracts. This finding is relevant given the biological relevance of the hydrogen-atom-transfer (HAT) reactions, and the use of peroxyl radicals, which are relevant to oxidative processes in biological system and food matrices, and is performed at pH and temperature conditions relatively close to physiological ones. Accordingly, Sadowska-Bartosz et al. [29] identified ORAC as a useful method for determining the antioxidant capacity of foods. However, this increase should not be interpreted as an overall improvement in antioxidant capacity, as DPPH and FRAP showed different responses to fermentation.

To further investigate the compositional changes underlying these responses, descriptive UPLC-DAD analysis was performed on the unfermented and 24 h fermented extracts. The chromatographic profile revealed differences in the distribution of individual phenolic compounds between unfermented and fermented samples, despite the relative stability of TPC. Among the compounds identified, kaempferol-3-*O*-glucoside, rutin and luteolin represented the main flavonoids detected in the unfermented material, although their abundance was affected by fermentation. Kaempferol-3-*O*-glucoside decreased following LP and LP + SC fermentation, while rutin was no longer detected after fermentation, under the analytical conditions. Luteolin was detected at low levels in the unfermented and LP-fermented samples but was not detected upon the LP + SC fermentation. These changes indicate a modification of the flavonoid fraction during fermentation. Although microbial glycosidase activity has been reported to promote the transformation of flavonoid glycosides during fermentation [30], the absence of the corresponding aglycones among the compounds detected and quantified in the present study prevents assigning these changes to a specific deglycosylation pathway. Fermentation also markedly affected the profile of hydroxycinnamic acids and caffeoylquinic acid derivatives. Chlorogenic acid decreased in fermented samples, whereas 3,4-*O*-caffeoylquinic acid and caffeic acid, which were not detected in the unfermented sample, became detectable following fermentation. The concomitant decrease in chlorogenic acid and appearance of other caffeoyl derivatives is compatible with microbial-mediated modification of the caffeoylquinic acid fraction, potentially involving hydrolytic and/or isomerisation processes, assessed in other plant matrices [11]. Interestingly, chicoric acid was detected in the unfermented sample but was no longer detected following fermentation, whereas 4-*O*-caffeoylquinic acid showed a less pronounced and fermented-dependent variation. Hydroxycinnamic acids, caffeic acid, chicoric acid, chlorogenic acid and their derivatives have been reported in both wild and cultivated chicory leaves, although their abundance can vary substantially depending on environmental conditions, agronomic practices, phenological stages, harvesting and dry procedures, as well as extraction methods [7,31,32,33]. Overall, the UPLC-DAD data indicate that fermentation reshaped the individual phenolic profile rather than causing a generalized depletion of phenolic compounds. This finding provides a possible explanation for the apparent discrepancy between the relative stability of TPC, and the changes observed in TFC and antioxidant activity. Indeed, a global assay such as TPC may remain relatively unchanged when the depletion of some compounds is accompanied by the formation, release, or increased extractability of others, even though the chemical properties of the resulting phenolic mixture may differ substantially. The differences in FRAP and ORAC responses occurred alongside changes in the phenolic profile. However, no quantitative correlation analysis or mechanistic experiments were performed to establish the contribution of individual compounds to these responses. Therefore, the observed antioxidant changes cannot be attributed to specific phenolic compounds on the basis of the present data.

The differences observed between fermentation with LP alone and in co-culture LP + SC further suggest that the composition of the starter culture may influence the extent and direction of these phytochemical changes. Although the two fermentations showed similar acidification kinetics and comparable TPC responses, some individual compounds displayed different concentrations according to the fermentation inoculum. In particular, the higher concentration of 3,4-*O*-caffeoylquinic acid and caffeic acid in LP + SC samples, together with the stronger depletion of chlorogenic acid, suggests that the presence of *S. cerevisiae* may have influenced the distribution and evolution of individual phenolic compounds during fermentation. Similar inoculum-dependent differences in individual phenolic compounds have been reported in mixed fermentations involving *S. cerevisiae* and lactic acid bacteria, supporting the view that starter composition can influence the extent and direction of phenolic changes during fermentation [11,34,35].

The findings support further investigation of fermented wild chicory leaves as a potential food ingredient. However, microbial safety, storage stability, sensory properties and performance following incorporation into food products remain to be evaluated. Further studies combining high-resolution LC-MS/MS, quantitative monitoring of individual metabolites throughout fermentation and bioaccessibility assessments are needed to clarify the mechanisms and nutritional relevance of the observed changes.

## 5. Conclusions

Fermentation of wild chicory leaves with *L. plantarum*, alone or in co-culture with *Saccharomyces cerevisiae*, was associated with acidification within 24 h and broadly unchanged total phenolic content. TFC, DPPH, FRAP and ORAC varied significantly with fermentation time, whereas neither the fermentation treatment nor its interaction with time had a significant effect. Descriptive UPLC-DAD analysis of samples indicated changes in individual phenolic profiles but could not establish biological treatment differences These findings highlight the value of complementary antioxidant assays when evaluating fermentation-related changes. Despite the exploratory nature and limitations of the present study, this work provides an initial investigation of the fermentation of locally collected wild chicory, an underexploited spontaneous plant resource. Further research integrating more extensive phytochemical characterisation with bioavailability, nutritional and functional studies is warranted to better elucidate the potential of fermented wild chicory leaves as a food ingredient.

## Figures and Tables

**Table 1:** UPLC-DAD qualitative parameters for the identification of reference compounds in the leaf chicory extracts. Rt, retention time (min; mean ± SD).

Chemical Class	Common Name	Rt	Data UV (λmax, nm)				Literature Data UV (λmax, nm) [18]
flavonol	quercetin	8.57 ± 0.15	210.0	256.6	364.0		210.0, 255, 269 sh, 301 sh, 370
flavon-*O*-glucoside	apigenin-7-*O*-glucoside	7.52 ± 0.09	210.0	266.4	336.7		210.0, 268, 333
flavonol	kaempferol	10.01 ± 0.10	221.5	266.2	364.0		221.5, 266, 294 sh, 322 sh, 367
flavon	apigenin	9.69 ± 0.11	213.3	266.4	336.7		213.3, 267, 296 sh, 336
flavon	luteolin	9.68 ± 0.15	212.0	252.2	347.5		212.0, 253, 267, 291 sh, 349
hydroxycinnamic acid	chicoric acid	6.93 ± 0.16	218.0	241.0 sl *	298.6 sh *	329.5	215.8, 328.4
flavonol-*O*-glucoside	kaempferol-3-*O*-glucoside	7.10 ± 0.15	212.2	265.2	347.5		212.2, 264, 301 sh, 350
hydroxycinnamic acid	3,4-*O*-caffeoylquinic acid	7.79 ± 0.09	218.0	242.9 sl	293.2 sh	327.1	250, 323, 303 sh
hydroxycinnamic acid	4-*O*-caffeoylquinic acid	6.33 ± 0.08	214.4	240.9 sl	295.2 sh	327.1	252, 324, 303 sh
hydroxycinnamic acid	chlorogenic acid	6.09 ± 0.11	215.6	245.5 sl	298.4 sh	325.9	218, 238 sl, 300 sh, 326
hydroxycinnamic acid	caffeic acid	6.46 ± 0.12	215.6	243.0 sl	296.2 sh	323.5	220, 240 sl, 295 sh, 325
flavonol-*O*-diglucoside	rutin	6.67 ± 0.10	210.0	255.6	354.7	484.9	204, 256, 355

* “sl” and “sh” referring to “shoulder” and “sharp line”, respectively.

**Table 2:** Calibration curve, R^2^, and linearity range for the identified compounds.

Compound	Calibration Curve	R^2^	Linearity Range (μg/mL)
luteolin	y = 3.9394x − 0.2804	0.9847	1.6–32.0
chicoric acid	y = 10.481x − 0.827	0.9976	1.9–30.0
kaempferol-3-*O*-glucoside	y = 3.0139x + 0.0937	0.9993	2.5–45.0
3,4-*O*-caffeoylquinic acid	y = 32.593x + 0.4188	0.9868	3.1–37.0
4-*O*-caffeoylquinic acid	y = 7.9703x − 0.0011	0.9984	2.5–24.8
chlorogenic acid	y = 2.8107x − 0.012	0.9948	1.0–23.6
caffeic acid	y = 0.7571x + 0.0039	0.9996	1.7–20.2
rutin	y = 0.771x − 0.0243	0.9952	2.5–20.0

**Table 3:** Total phenolic content (TPC), total flavonoid content (TFC), DPPH radical scavenging capacity, FRAP reducing capacity and ORAC antioxidant capacity of wild chicory leaf fermented with *L. plantarum* (LP) or *L. plantarum* + *S. cerevisiae* (LP + SC), for 24 and 48 h, compared with unfermented material (T0). Data are expressed as mean ± SD of two independent biological batch per condition. Technical triplicates were averaged within each batch before calculating these summaries. Values sharing a letter do not differ significantly in time-point comparisons (*p* < 0.05). ns *p* > 0.05, * *p* < 0.05, ** *p* < 0.01.

		TPC	TFC	DPPH	FRAP	ORAC
		mg AGE/g	mg CE/g	mg TE/g	µmol Fe^2+^E/g	μmol TE/g
**Fermentation**	**LP**	3.547 ± 0.128	7.807 ± 0.538	1.007 ± 0.073	32.008 ± 2.410	37.984 ± 3.639
	**LP + SC**	3.650 ± 0.281	7.932 ± 0.673	0.897 ± 0.043	32.849 ± 1.432	39.192 ± 2.405
	***sig***	ns	ns	ns	ns	ns
**Time**	**T0**	3.795 ± 0.040	9.437 ± 0.000 ^a^	0.909 ± 0.068 ^b^	36.970 ± 0.000 ^a^	31.570 ± 4.073 ^b^
	**T1**	3.629 ± 0.369	6.994 ± 0.632 ^b^	1.095 ± 0.160 ^a^	33.189 ± 3.781 ^ab^	42.191 ± 1.215 ^a^
	**T2**	3.372 ± 0.248	7.178 ± 0.883 ^b^	0.853 ± 0.062 ^b^	27.126 ± 1.989 ^b^	42.003 ± 3.058 ^a^
	***sig***	ns	**	**	*	**
**Fermentation × Time**	**LP × T0**	3.795 ± 0.049	9.437 ± 0.000	0.909 ± 0.083	36.970 ± 0.000	31.570 ± 4.988
	**LP × T1**	3.541 ± 0.371	6.912 ± 0.577	1.218 ± 0.123	32.258 ± 3.907	41.573 ± 1.354
	**LP × T2**	3.306 ± 0.061	7.073 ± 1.037	0.895 ± 0.014	26.795 ± 3.323	40.810 ± 4.575
	**LP + SC × T0**	3.795 ± 0.049	9.437 ± 0.000	0.909 ± 0.083	36.970 ± 0.000	31.570 ± 4.988
	**LP + SC × T1**	3.717 ± 0.490	7.077 ± 0.915	0.972 ± 0.019	34.120 ± 4.914	42.810 ± 1.032
	**LP + SC × T2**	3.439 ± 0.404	7.283 ± 1.103	0.811 ± 0.066	27.458 ± 0.619	43.195 ± 1.195
	***sig***	ns	ns	ns	ns	ns

*“sig”* referring to significance.

**Table 4:** Qualitative and quantitative profile of phenolic compounds identified by UPLC-DAD in unfermented and 24 h fermented chicory leaf extracts obtained with *L. plantarum* (LP) and *L. plantarum* + *S. cerevisiae* (LP + SC). Compounds were identified based on their retention times (Rt) compared with standards and quantified as mg/100 g of lyophilised samples. Results are reported as mean ± SD of three analytical replicates of a single pooled sample per condition, prepared by combining material from the two biological batches. The SD reflects analytical variability and does not estimate variability between biological batches. Comparisons between pooled samples are descriptive; no inferential statistical tests were performed. n.d., not detected; tr., traces, < than the lower level of linearity range for each standard in the analytical samples.

		mg/100 g		
	Rt	Unfermented	LP	LP + SC
quercetin	8.57 ± 0.15	n.d.	n.d.	n.d.
apigenin-7-*O*-glucoside	7.52 ± 0.09	n.d.	n.d.	n.d.
kaempferol	10.01 ± 0.10	n.d.	n.d.	n.d.
apigenin	9.69 ± 0.11	n.d.	n.d.	n.d.
luteolin	9.68 ± 0.15	6.09 ± 0.01	7.13 ± 0.09	n.d.
chicoric acid	6.93 ± 0.16	8.13 ± 0.45	n.d.	n.d.
kaempferol-3-*O*-glucoside	7.10 ± 0.15	40.68 ± 0.01	19.08 ± 0.46	11.78 ± 0.02
3,4-*O*-caffeoylquinic acid	7.79 ± 0.09	tr.	14.03 ± 0.09	22.10 ± 1.04
4-*O*-caffeoylquinic acid	6.33 ± 0.08	15.27 ± 0.60	18.53 ± 3.25	11.18 ± 0.19
chlorogenic acid	6.09 ± 0.11	66.70 ± 0.54	19.53 ± 0.36	4.92 ± 0.30
caffeic acid	6.46 ± 0.12	tr.	7.77 ± 1.37	16.25 ± 1.64
rutin	6.67 ± 0.10	66.08 ± 0.43	n.d.	n.d.

## Data Availability

The raw data supporting the conclusions of this article will be made available by the authors on request.
